# Long-term field study reveals that warmer summers lead to larger and longer-lived females only in northern populations of Natterer’s bats

**DOI:** 10.1007/s00442-023-05318-9

**Published:** 2023-02-11

**Authors:** Bianca Stapelfeldt, Christoph Tress, Ralf Koch, Johannes Tress, Gerald Kerth, Alexander Scheuerlein

**Affiliations:** 1 Zoological Institute and Museum, University of Greifswald, Greifswald, Germany; 2 Fledermausforschungsprojekt Wooster Teerofen e.V., Wooster Teerofen, Germany; 3 Naturpark Nossentiner/Schwinzer Heide, Plau am See OT Karow, Germany

**Keywords:** Climate change, Global warming, Survival, Body size, Bergmann's rule, Temperature

## Abstract

Animals often respond to climate change with changes in morphology, e.g., shrinking body size with increasing temperatures, as expected by Bergmann’s rule. Because small body size can have fitness costs for individuals, this trend could threaten populations. Recent studies, however, show that morphological responses to climate change and the resulting fitness consequences cannot be generalized even among related species. In this long-term study, we investigate the interaction between ambient temperature, body size and survival probability in a large number of individually marked wild adult female Natterer’s bats (*Myotis nattereri*). We compare populations from two geographical regions in Germany with a different climate. In a sliding window analysis, we found larger body sizes in adult females that were raised in warmer summers only in the northern population, but not in the southern population that experienced an overall warmer climate. With a capture-mark-recapture approach, we showed that larger individuals had higher survival rates, demonstrating that weather conditions in early life could have long-lasting fitness effects. The different responses in body size to warmer temperatures in the two regions highlight that fitness-relevant morphological responses to climate change have to be viewed on a regional scale and may affect local populations differently.

**Supplementary Information:**

The online version contains supplementary material available at 10.1007/s00442-023-05318-9.

## Introduction

Body size is a trait that has often been described to be affected by global warming (e.g., Chamaillé-Jammes et al. 2006; Daufresne et al. 2009; Mundinger et al. 2021). Some studies suggest that changes in body size may follow Bergmann’s rule with increasing temperatures leading to reduced body sizes (Daufresne et al. 2009; Yom-Tov et al. 2010) as an adaptation to warmer environments because the larger surface area to volume ratio leads to heat loss (Bergmann 1847). On the other hand, the large individual body size is often linked to high individual fitness, specifically to increased fecundity (Chamaillé-Jammes et al. 2006; Ozgul et al. 2010; Hernández‐Pacheco et al. 2021). Shrinking body size in response to climate change could therefore be maladaptive and thus threaten populations of species where large body size is connected to higher individual fitness and thus ultimately population persistence. As a consequence, body size changes in response to global warming and the consequences for population persistence received growing research interest during recent years. However, not all species show shrinking body size in response to global warming, with several studies finding increasing body sizes or no change in body size (Gardner et al. 2011; Nengovhela et al. 2020). In a meta-analysis of 50 rodent species across the world, 29 species showed no change in body size, eight species increased and 13 species decreased in body size in response to global warming (Nengovhela et al. 2020). In conclusion, responses of body size to rising ambient temperatures are species-specific and cannot be generalized.

One critical aspect which is often linked to body size and the associated fitness effects are early life conditions (Lindström 1999; Metcalfe and Monaghan 2001; Meniri et al. 2019; Mundinger et al. 2021). Time of birth, hatching date, and parental provisioning of nutrients during pre- and postnatal stages can all affect growth rates and early development (Daan et al. 1990; Richner 1992; Metcalfe and Monaghan 2001; Meniri et al. 2019). At the same time, reproductive phenology and the availability of resources are highly sensitive to weather patterns. This can be observed, e.g., in many bird species that started breeding earlier in warmer springs and thereby matched nestling time with the peak in prey abundance (e.g., Both et al. 2006; Shave et al. 2019; Wesołowski et al. 2021). The fitness effects of beneficial early life conditions then often have long-term fitness consequences, e.g., affecting survival (Daan et al. 1990; Chamaillé-Jammes et al. 2006; Frick et al. 2010), reproduction (Ransome 1989; Chamaillé-Jammes et al. 2006; Linton and Macdonald 2018) or the acquisition of crucial resources such as breeding territories (Richner 1992).

Bats of the temperate zone also show phenological shifts such as earlier birth dates at warmer spring temperatures (Reiter 2004; Lučan et al. 2013; Mundinger et al. 2021). Being small, and without extensive fat reserves in summer, bats are income breeders. This means that female bats compensate for increased energy expenditure during reproduction by increased food intake, and therefore heavily rely on foraging success, as opposed to capital breeders, that fuel reproduction using previously accumulated body reserves (Henry et al. 2002; Matheson et al. 2010; Encarnação et al. 2012; Culina et al. 2019). In contrast to the life-history hypothesis which predicts that small mammals have short life spans and high reproductive output, bats are extremely long-lived with long generation times (Wilkinson and South 2002). Being long-lived, bats may not be able to quickly respond through genetic adaptation to climate change but show a high degree of plasticity to cope with aversive weather conditions. Plastic responses to aversive weather conditions include behaviors such as social thermoregulation and roost switching as well as physiological responses such as torpor (Kerth et al. 2001; Pretzlaff et al. 2010; Geiser 2013; Doty et al. 2016).

During torpor, body temperature and metabolic rate are reduced, which allows bats to save energy during adverse conditions (Geiser 2004). However, entering a torpor during the reproductive period also incurs costs, which can be linked to late parturition, reduced milk production and slow juvenile growth rates (Wilde et al. 1999; Reiter 2004; Linton and Macdonald 2018). As a result, cold temperatures during juvenile growth can be associated with smaller adult body size (Tuttle 1976; Zahn 1999; Mundinger et al. 2021). Therefore, if conditions are not too cold, reproductive females avoid long torpor bouts that would compromise juvenile development and instead minimize energy expenditure by roosting in larger groups which allows for improved thermoregulation (Willis and Brigham 2007; Pretzlaff et al. 2010). The effect of social thermoregulation on body size was shown in Bechstein’s bats (*Myotis bechsteinii*), where individuals that grew up in larger colonies reached a larger body size than individuals that grew up in smaller colonies (Mundinger et al. 2021). However, this effect was not observed in the species *Myotis myotis*, which is typically living in much larger colonies than Bechstein’s bats (Zahn 1999; Mundinger et al. 2021). Overall, the available data suggest that large colony sizes have the potential to buffer the adverse influence of cold weather bouts.

The beneficial effect of fast juvenile development in bats, leading to an early onset of flight, early maturity and an increased time span to gather the fat reserves needed for the first hibernation, is well known (Frick et al. 2010; Linton and Macdonald 2018, 2020). However, the effect of a large adult body size on survival and reproduction is less clear. Some studies suggest advantages of large body size in female bats for reproduction (Myers 1978; Findley 1979; Ransome 1989; Mundinger et al. 2022; Stapelfeldt et al. 2022), whereas other studies found higher mortality in larger individuals (Fleischer et al. 2017; Mundinger et al. 2021). The interactions between weather conditions, body size and fitness in bats cannot be generalized because responses can vary between species and even within species on a spatiotemporal scale (Nengovhela et al. 2020; Alston et al. 2021). An analysis of 20 North-American bat species revealed that intraspecific variation in body size followed resource availability across time (larger individuals in years with higher insect densities) but followed Bergmann’s rule across space (larger individuals in higher latitudes) (Alston et al. 2021). In conclusion, the available studies underline the importance of considering intraspecific variation in body size not only on a temporal but also on a spatial scale.

Here, we study the effect of ambient temperature on the adult body size of female Natterer’s bats (*Myotis nattereri*) from two regions in Germany and assess how the individual survival of adult females is affected by body size. Natterer’s bats are, like all European bat species, strictly protected by law. This medium-sized species (ca. 7–10 g, Dietz et al. 2016) enters hibernation relatively late in autumn compared to other European bat species and shows some foraging activity in the winter season (Hope and Jones 2012; Hope et al. 2014; Meier et al. 2022). Mating occurs in autumn and winter and Natterer’s bats emerge from hibernation between January and April (Reusch et al. 2019; Meier et al. 2022). Female Natterer’s bats give birth to one offspring per year between June and July (Linton and Macdonald 2018). Maternity colonies of Natterer’s bats comprise up to 80 adult females and form from April to October (Zeus et al. 2018).

Recently, a study on Bechstein’s bats revealed that ambient temperatures during the period of juvenile growth in summer strongly influenced adult body size, with higher temperatures leading to larger individuals (Mundinger et al. 2021). In the first part of our study, we follow the approach of Mundinger et al. (2021) and investigate with a sliding window analysis if there is a sensitive time window, during which ambient temperatures influence juvenile growth in Natterer’s bats from two different regions. One region is located in northern Germany with a cooler climate than the other region, located in southern Germany. Based on the results of the related Bechstein’s bat, we expect that in Natterer’s bats, ambient temperatures during the juvenile growth period are positively correlated with adult body size. However, we expect that the effect could be buffered by higher summer temperatures in the southern region and, therefore, being more visible in the northern region.

In a second analysis, we apply a capture-mark-recapture analysis based on a Cormack Jolly Seber model approach to investigate drivers for survival. In Bechstein’s bats, a large body size was linked to higher mortality (Fleischer et al. 2017; Mundinger et al. 2021). We thus tested the effect of *body size* on survival in our study populations. Energy expenditure and thus costs for reproduction during summer may be lower in the warmer region and could also vary between years depending on the weather conditions. Because of a link between reproductive effort and survival in Bechstein’s bats (Mundinger et al. 2022), we tested if the survival probability of adult female Natterer’s bats differs by *region* (north vs. south), and *year* in our study populations*.* Moreover, we assessed the relative importance of *mean colony size* on survival, because individuals that live in larger colonies could make more effective use of thermoregulation (Willis and Brigham 2007)*.*

## Material and methods

### Study site and data collection

We analyzed data from two different regions where bat and bird boxes had been provided for the local bat populations for roosting: one box area in Bavaria (*WB*, southern Germany) near the city of Würzburg (WB) and three box areas in Mecklenburg-Western Pomerania (*TUP, WT, Bossow,* northern Germany) in the Nature-Park Nossentiner/Schwinzer Heide (NSH). In Bavaria, data from Natterer’s bats were collected since 2011 and in Mecklenburg-Western Pomerania since 1990. Each year in May, and again between the end of July and September after the juveniles had fledged, boxes occupied by Natterer’s bats were carried to the field stations, which are located close to the box areas in each region, where handling took place. During the handling of the bats, forearm length (FAL) was measured and age (juvenile versus adult) was assessed. Juveniles were identified by unfused metacarpal-phalangeal epiphyseal gaps (Hoying and Kunz 1998). To ensure that only the size of mature individuals is analyzed, we used only forearm measurements of the first re-capture as an adult after an individual’s first hibernation. In the NSH region, all unmarked bats received a forearm band and since 2012 individuals were additionally marked with a subcutaneously implanted RFID-Tag (Trovan, Germany) in the box areas TUP and Bossow. In WB all unmarked females were marked with RFID-Tags exclusively. Capture and handling procedure took place during the daytime. After measurements were taken, the bats were released to their original box and the boxes were brought back to their original place in the forest before the evening.

### Sliding window analysis of the effects of weather on body size

Statistical analysis was performed with the program R (R core Team 2020). To investigate the influence of ambient temperatures during the period of juvenile growth on FAL reached as an adult, we applied a sliding window analysis with the package climwin (Bailey and van de Pol 2016). In this approach, models with all possible time windows are fitted and compared using the information-theoretic model selection criteria AICc and the percentage of models contained in the 95% confidence set for each tested weather parameter (Bailey and van de Pol 2016; van de Pol et al. 2016). For the AICc criterion, the difference between the fitted climate model to the null model is calculated (ΔAICc). The value of the 95% confidence set indicates the percentage of models that each explain 95% of the response, out of all models fitted in the analysis. If the percentage of models contained in the set is low, we can be confident that the analysis reveals a real climate signal. If the percentage of models in the set is high, a large number of models containing different time windows that are equally likely, indicating the absence of a specific climate signal (Bailey and van de Pol 2016; van de Pol et al. 2016). We obtained climate data from the archive of the DWD meteorological station Würzburg (ID 05705) for the WB population and Goldberg (ID 01694) for the NSH population. We analyzed the effect of daily minimum, mean and maximum temperatures during the birth year on adult FAL separately for each region. For this analysis, we excluded all individuals with unknown birth year as well as all individuals that did not survive the first hibernation (no adult FAL available). This resulted in a dataset of 9 years (2012–2020, no juveniles were marked in WB in 2011) with a total of *N* = 112 individuals from WB and 30 years (1990–2020) with a total of *N* = 520 individuals from NSH. Following the analysis of Mundinger et al. (2021), we designed the sliding window approach to start on May, 1st and end on September 30th. As null-model we fitted the intercept model. To verify that the different time spans and sample sizes for WB and NSH did not affect the results, we randomly drew for each year (2012–2020) as many females from the NSH data as were present in the specific year in the WB data, and repeated the climwin analysis for the restricted NSH dataset (results see Supplemental Table S1 and Fig. S1). Results of the main analysis and of the analysis with the restricted NSH dataset were verified by 100 randomizations.

### Survival analysis

In the next step, we investigated influences on adult survival with live encounter models (Cormack–Jolly–Seber models) with the package *marked* (Laake et al. 2013). For this analysis, capture data are transformed to capture histories. If an individual was captured at least once during a year, this is represented with a ‘1’ and with ‘0’ if an individual was not captured in the respective year. We chose maximum likelihood estimates for survival (*ɸ*) and recapture (*p*) (Lebreton et al. 1992). As a start, we applied goodness of fit analyses to the data with the program R2UCARE (Gimenez et al. 2018). Test 2ct was significant for all box areas in NSH but not for WB, indicating heterogeneity in recapture rates in NSH, but not in WB. Individuals of colonies that live on the edge of the box areas are probably less often captured than individuals of colonies living in the core of the box areas. Based on the knowledge that individuals roosted together in a box, we used the program Gephi and the implanted modularity function for colony identification (Blondel et al. 2008).

We identified six regularly recaptured colonies and several sporadically captured colonies (edge groups) in the NSH region and one single colony in WB. We excluded edge groups from the data and then computed the variance inflation indicating a moderate level of overdispersion (c-hat = 1.5). We then fitted the survival models testing *region* (WB vs. NSH), *box area* (WB, TUP, WT, Bossow), *year* and *effort* (how many capture-events per year and box area), their additive effects and interactions for recapture probability (*p*). For the survival term (*ɸ*) we modelled the effect of *year, FAL*, *region* (WB vs. NSH) and the *mean colony size* of each colony (for details see Supplemental Section *colony size*), the additive effects and the interaction of these variables.

Because the exact birth year is not a relevant predictor in this analysis, we also included all individuals with unknown birth year. This resulted in a dataset of 1177 individuals. In the last step, we adjusted the models for overdispersion and computed the QAIC for model selection.

## Results

**Fig. 1:**
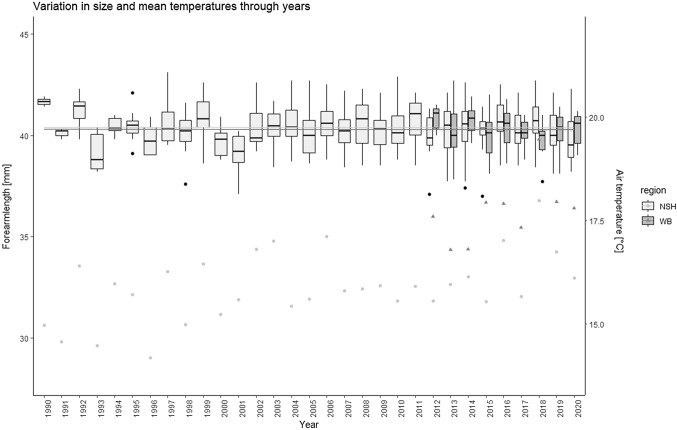
Boxplots show the variation of forearm length (mm, left axis) of adult Natterer’s bat females from the region WB (2012–2020, dark grey) and NSH (1990–2020, light grey). Scatter plots display the mean summer ambient temperature (May, 1st–September 30th) in the two regions WB (triangles) and NSH (dots)

**Fig. 2:**
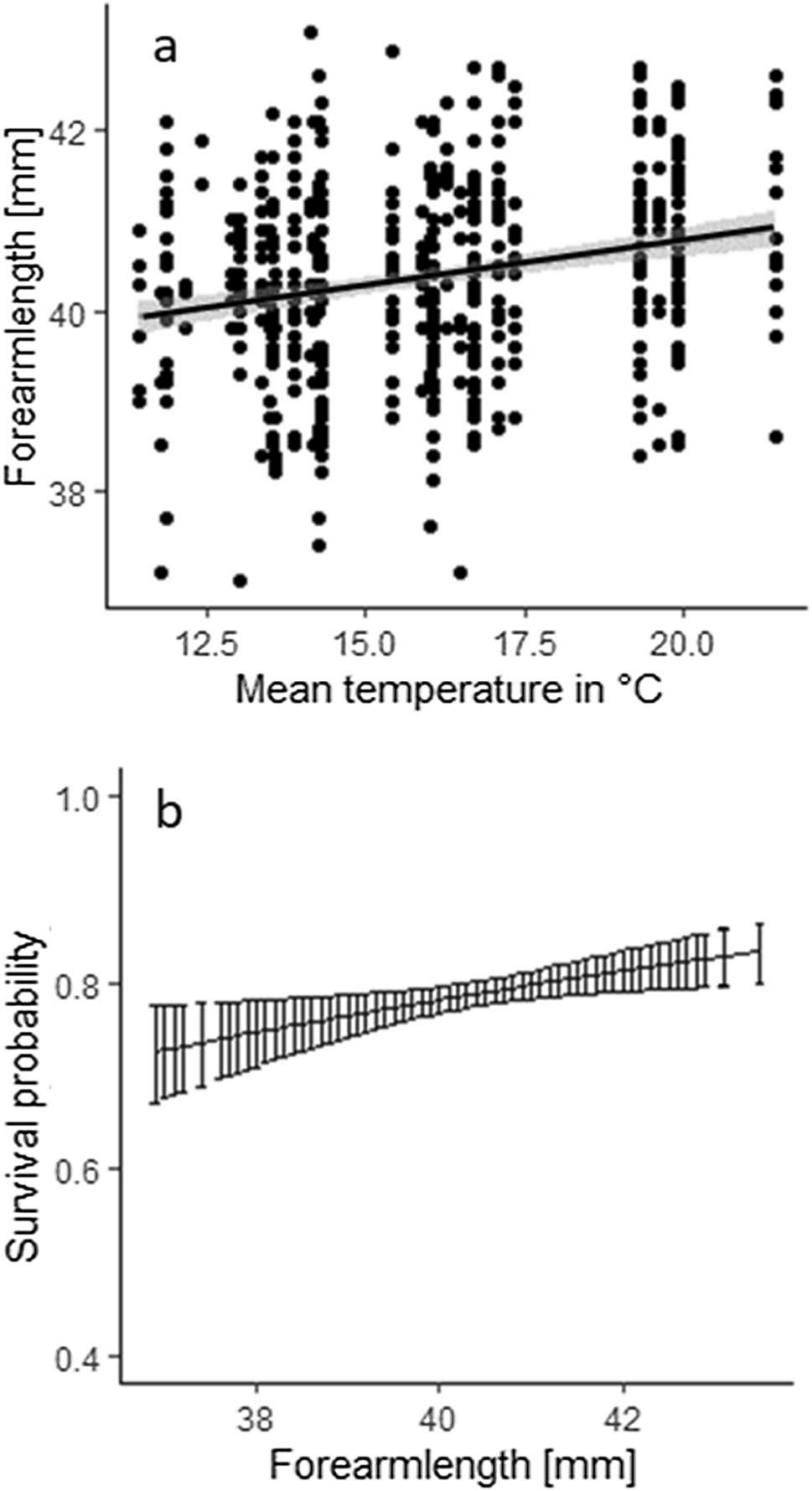
**a** Predicted response of the best-supported model for mean temperatures. Forearm length (mm) of females in NSH increases with warmer mean summer temperatures (shaded area defines the 95% confidence intervals). **b** Predicted survival probability for adult Natterer’s bats of both regions (NSH and WB) as a function of forearm length (mm) (error bars show the 95% confidence intervals)

Between 2012 and 2020 (time period for which we analyzed data of both areas) mean summer temperatures (May, 1st to September 30th) in WB were 1.3 °C warmer than in NSH (Tmean_WB_ = 17.6 °C, Tmean_NSH_ = 16.3 °C, Fig. 1, Fig. S2). Mean FAL of individuals from NSH_1990–2020_ was 40.4 (± 1.1) mm (NSH_2012–2020_ = 40.4 (± 1.1)) and thus very similar to the mean FAL from individuals from WB_2012–2020_ 40.3 (± 1.0) mm (Fig. 1). In the climwin analysis, the effect of mean temperatures on FAL was best supported for Natterer’s bats from NSH where higher mean ambient temperatures led to larger individuals by + 0.2 mm per °C (best model: ∆AICc = − 28.12, *p* < 0.001, *R*-squared: 0.06, Fig. 2A). Model averaging revealed a relatively large time span (median window: May, 23rd—September 14th), where body size of Natterer’s bats was sensitive to mean ambient temperatures (models falling into 95% confidence set: 25%) (see Supplemental Table S1). Results from a reduced NSH dataset corresponding in sample size and range of years to the WB data (112 individuals, years 2012 until 2020) confirmed the above results (see Supplemental Table S1 and Fig. S1). For the analysis of the WB population, the low ∆AICc values for the best models (< 10) and the high proportions of models contained in the 95% confidence set for all tested variables (56–66%) indicated no clear climate effect on FAL (see Supplemental Table S1).

For the survival analysis, 110 different models were fitted. We therefore only present the ten best models in Table 1 (see Supplemental Table S2 for all models and Table S3 for model parameter estimates). The best-supported model included the interaction term between region and year for recapture probability (*p*) (see Supplemental Table S4) and the parameter FAL for survival (*ɸ*). Larger adult females had higher survival probabilities than smaller adult females (2% higher survival probability by 1 mm increase in FAL) (Fig. 2B, Supplemental Table S5).

**Table 1:** Results of the ten best models from the survival analysis

Model nr.	Model	npar	QAIC
1	**ɸ(~ FAL)*p*(~ year** * **region)**	45	3109.91
2	**ɸ**(~ region + FAL)***p***(~ year * region)	46	3111.435
3	**ɸ**(~ FAL + colony size)**p**(~ year * region)	46	3111.548
4	**ɸ**(~ region)**p**(~ year * region)	45	3112.809
5	**ɸ**(~ colony size)***p***(~ year * region)	45	3112.943
6	**ɸ**(~ region * FAL)***p***(~ year * region)	47	3113.285
7	**ɸ**(~ FAL * colony size)***p***(~ year * region)	47	3113.309
8	**ɸ**(~ FAL)***p***(~ year + box area)	37	3135.549
9	**ɸ**(~ region + FAL)***p***(~ year + box area)	38	3137.294
10	**ɸ**(~ FAL + colony size)***p***(~ year + box area)	38	3137.443

Survival probability (*ɸ*): FAL = forearm length, colony size = mean colony size, year = year of observation, region = WB vs. NSH; recapture probability (*p*): year = year of recapture, region = WB vs. NSH; npar = number of parameters in the model, QAIC = AIC adjusted for overdispersion (c-hat 1.5)

## Discussion

We found larger adult body sizes (FAL) in response to higher mean summer temperatures in female Natterer’s bats from the northern region (NSH), but not in bats from the southern region (WB). It is important to note that the different responses of bats from the two regions are not caused by the different time spans of analyzed data (NSH since 1990, WB since 2012). When we restricted the analyzed dataset from NSH to the same time period as WB (since 2012), we still found the positive effect of ambient temperatures on FAL in NSH. We assume, that the reason for the different results (positive effect in NSH and no effect in WB) could be explained by the regional differences in mean summer temperatures. With exception of the very warm summer in 2018, the highest mean temperatures in NSH almost reach the level of the lowest mean summer temperatures in WB. It is plausible, therefore, that the overall warmer summer temperatures in WB buffered the effect of summer temperatures on FAL because even relatively cold summers in WB were still warm enough for juvenile Natterer’s bats do not show reduced growth.

The positive effect of ambient temperature on FAL found in the northern Natterer’s bat population was not very strong (explained only 6% of the response) and was much weaker than the respective positive effect found in Bechstein’s bats (compare Mundinger et al. 2021). Interestingly, the Bechstein’s bat populations that exhibit a strong correlation between ambient temperatures and FAL (Mundinger et al. 2021), live in the same region (WB) as the southern population of Natterer’s bats from this study, where we found no such effect. This highlights the fact that even closely related species that are living in the same region can show species-specific responses to warmer environmental conditions. Possibly species ranges of the two Myotis species might help to explain their different sensitivity to ambient temperature. The northern border of Natterer’s bats range is at higher latitudes than the northern border of Bechstein’s bats (Dietz et al. 2016). This suggests that Natterer’s bats can probably better cope with cool weather conditions than Bechstein’s bats, which is reflected e.g. in the activity and foraging behavior of Natterer’s bats during winter (Hope and Jones 2012; Hope et al. 2014). Furthermore, in our study site WB, Natterer’s bats form larger colonies than Bechstein’s bats (Zeus et al. 2018; Mundinger et al. 2021). This might enable them to make more effective use of social thermoregulation and thus become more independent from ambient temperatures (Willis and Brigham 2007).

Most interestingly, and not shown before in bats, is the positive effect of large body size on individual survival in our study. Variation in body size can be caused by intrinsic (genetic, e.g. Mousseau and Roff 1987) and extrinsic (environmental conditions during early life, Ransome 1989; Zahn 1999; Mundinger et al. 2021) factors. In our study, we can only consider extrinsic factors, which indicate that large body size is connected to warm summer temperatures (Ransome 1989; Zahn 1999; Mundinger et al. 2021). Warm temperatures allow for early birth dates and fast juvenile growth and thus can lead to higher survival probabilities of young bats (Ransome 1989; Hoying and Kunz 1998; Frick et al. 2010). Early parturition and fast juvenile development probably increases the timespan available for juveniles to build sufficient fat reserves prior to first hibernation and therefore potentially increases winter survival (Frick et al. 2010). Here, we only analyzed the effect of the body size of adult individuals that survived the first hibernation. Because individuals which are born late or grew up under inclement weather conditions show both, smaller body size (Ransome 1989; Zahn 1999; Mundinger et al. 2021) and lower survival rates (Ransome 1989; Frick et al. 2010), the smallest individuals likely died before their first emergence from hibernation. We, therefore, assume that the link between body size and survival would be even stronger if we had also taken into account the individuals that did not survive their first winter. The fact, that we still find this pattern in individuals that survived their first hibernation, shows that costs or benefits of early life conditions have long lasting fitness effects (Ransome 1989; Meniri et al. 2019).

In hibernating mammals, including our study species, adult summer survival is typically lower than winter survival (Turbill et al. 2011; Reusch et al. 2019). Here, we only analyzed data from females, so probably survival costs may be connected with reproductive effort. Costs of reproduction may be lower in larger females because of the proportional reduction of the wing load during pregnancy or when they carry their offspring or reduced relative costs of milk production (Myers 1978). Moreover, larger females may have energetically benefits in maintaining homeothermy (Findley 1979; Ransome 1989). Interestingly, in Bechstein’s bats larger females reproduce faster than smaller individuals, which explains the lower survival in larger females (Mundinger et al. 2022). However, this trade-off between fast reproduction and survival does not appear to exist in Natterer’s bats, where larger females also reproduce faster (Stapelfeldt et al. 2022), but at the same time have a higher survival (this study).

Our results suggest that Natterer’s bats might profit from increased temperatures caused by climate change. However, the effect of rising temperatures could affect populations in different parts of the species’ distribution differently: while the northern populations might profit by an increase in temperatures, a temperature rise in the southern region at some point might exceed the heat tolerance limit if maternity roosts overheat (Lourenço and Palmeirim 2004; Crawford and Keefe 2021) or drought caused water stress in reproducing females (Adams and Hayes 2008; Adams 2010; Amorim et al. 2015). The fact that in Bechstein’s bats the effect of large body size in response to warm temperatures on survival was negative (Mundinger et al. 2021) highlights that fitness consequences in response to temperature rise can go in opposite directions even in closely related species. Taken together, these findings clearly demonstrate that the interactions between environment, phenotypic traits and fitness are highly species specific in bats and have to be considered not only on a temporal but also on a spatial scale (Fleischer et al. 2017; Gardner et al. 2017; Nengovhela et al. 2020; Alston et al. 2021; Mundinger et al. 2021).

## Conclusion

Our findings suggest that warm weather conditions experienced early in life have long lasting positive fitness effects because they lead to larger body sizes. As population dynamics in hibernating mammals are mainly driven by summer mortality (Turbill et al. 2011; Reusch et al. 2019), we assume that the observed survival advantages in our study result from energetic benefits for larger females during reproduction (Myers 1978; Findley 1979; Ransome 1989). The positive effect of warmer summer temperature on body size and thus indirectly on survival implies that northern Natterer’s bat populations might benefit from global warming. However, our results also indicate that populations in warmer regions might not profit from global warming in the same way. Thus, our findings raise the demand for further investigations on regional differences in morphological responses to global warming to better understand the implications of climate change on population persistence in vulnerable species such as bats.

## Supplementary Information

Below is the link to the electronic supplementary material.

Supplementary file1 (DOCX 621 KB)

## Data Availability

The datasets used and/or analyzed during the current study are available from the corresponding author upon reasonable request.

## Code availability

The code used to analyze the data is available from the corresponding author upon reasonable request.
