# Supplementary material for: Long-term field study reveals that warmer summers lead to larger and longer-lived females only in northern populations of Natterer’s bats
Source: Oecologia. 2023 Feb 11;201(3):853–61. doi: 10.1007/s00442-023-05318-9 (PMC10038953; doi:10.1007/s00442-023-05318-9)
Supplement: Supplementary file 1 — Supplementary file1 (DOCX 621 KB) [file 442_2023_5318_MOESM1_ESM.docx]

# Supplement

## climwin

**Table S1**: Results of the climwin-analysis for the influence of daily maximum temperature (T max), daily minimum temperature (T min) and mean daily temperature (T mean) during the birth year on the adult forearm length (FAL) on individuals from northern Germany (NSH) and southern Germany (WB). Function = tests for linear effect of the climate variable, ∆AICc = ∆AICc of the best model compared to the intercept model, best model window = sensitive time window of the best supported model (number of days before 30.09.), median window = sensitive time window of models averaged from the 95 % confidence set, model falling into 95 % confidence set = % of models that make up the top 95 % of model weights, range: analyzed time span.

| **Region** | **climate variable** | **function** | **∆AICc** | **best model window** | **median window** | **models falling into 95 % confidence set** | **range** |
| --- | --- | --- | --- | --- | --- | --- | --- |
| NSH  1990-2020  N=520 | T max | linear | -22.68 | 29-28 | 134-23 | 26 % | 01.05.-30.09. |
|  | T min | linear | -23.79 | 36-10 | 117-12 | 23 % | 01.05.-30.09. |
|  | T mean | linear | -28.12 | 20-20 | 131-17 | 25 % | 01.05.-30.09. |
| NSH reduced  2012-2020  N=112 | T max | linear | -16.84 | 141-141 | 147-81 | 16 % | 01.05.-30.09. |
|  | T min | linear | -13.40 | 11-11 | 134-56 | 43 % | 01.05.-30.09. |
|  | T mean | linear | -17.12 | 141-141 | 144-68 | 23 % | 01.05.-30.09. |
| WB  2012-2020  N=112 | T max | linear | -6.11 | 88-31 | 109-40 | 66 % | 01.05.-30.09. |
|  | T min | linear | -8.03 | 107-49 | 112-35 | 56 % | 01.05.-30.09. |
|  | T mean | linear | -7.14 | 103-54 | 111-41 | 64 % | 01.05.-30.09. |

Like in the analysis with the full dataset, the climwin analysis for the reduced NSH dataset from 2012-2020 revealed, that higher mean temperatures led to larger individuals (best model: by +0.31 mm per °C, ∆AICc = -17.12, p < 0.001, R-squared: 0.16, Fig. S1). However, the median time window where mean temperatures had an effect on FAL was shorter than in the analysis with the full dataset and ranged from May 9^th^ to July 24^th^.


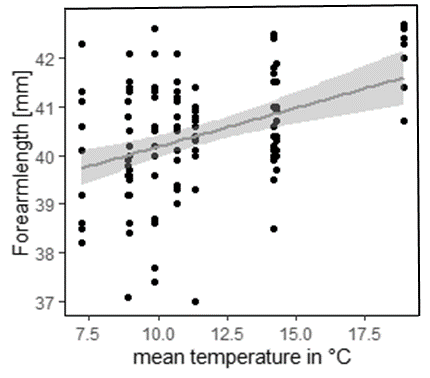


**Figure S1:** Results from the restricted NSH dataset: predicted response of the best supported model for mean temperatures. Forearm length (mm) increases with warmer mean summer temperatures also for females in the reduced NSH dataset (2012-2020).

## Temperatures through years

**
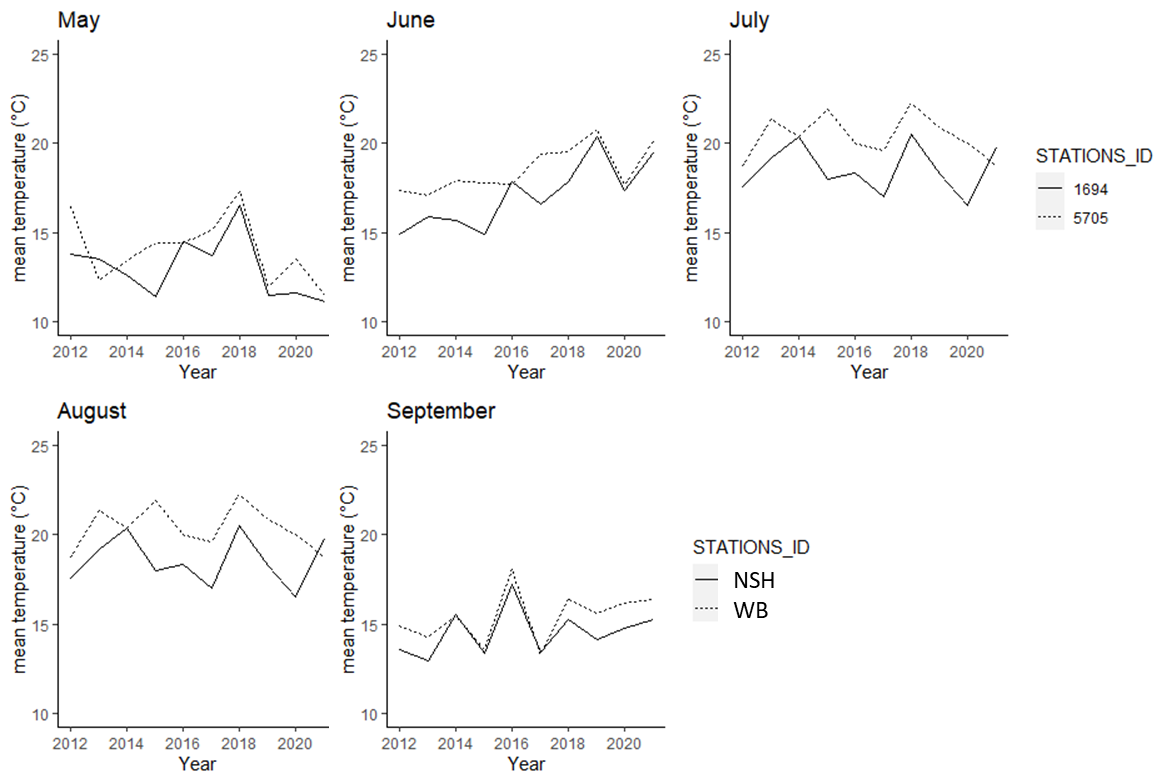
**

**Figure S2:** Mean temperatures during the investigated time window (May, 1^st^ – September 30^th^) in NSH (northern Germany, solid line) and WB (southerm Germany, scattered line).

## Survival analysis

**Tab. S2:** Results of the survival models: survival probability (*ɸ*): FAL = forearm length, colony size = mean colony size, year = year of observation, region = WB vs. NSH; recapture probability (*p*): year = year of recapture, region = WB vs. NSH, box area = TUP, WT, Bossow or WB, effort = how many capture-events per year and box area; npar=number of parameters in the model, QAIC = AIC adjusted for overdispersion (c-hat 1.5).

| model nr. | model | npar | QAIC |
| --- | --- | --- | --- |
| 1 | ɸ(~FAL)p(~year * region) | 45 | 3109.91 |
| 2 | ɸ(~region + FAL)p(~year * region) | 46 | 3111.435 |
| 3 | ɸ(~FAL + colony size)p(~year * region) | 46 | 3111.548 |
| 4 | ɸ(~region)p(~year * region) | 45 | 3112.809 |
| 5 | ɸ(~colony size)p(~year * region) | 45 | 3112.943 |
| 6 | ɸ(~region * FAL)p(~year * region) | 47 | 3113.285 |
| 7 | ɸ(~FAL * colony size)p(~year * region) | 47 | 3113.309 |
| 8 | ɸ(~FAL)p(~year + box area) | 37 | 3135.549 |
| 9 | ɸ(~region + FAL)p(~year + box area) | 38 | 3137.294 |
| 10 | ɸ(~FAL + colony size)p(~year + box area) | 38 | 3137.443 |
| 11 | ɸ(~region)p(~year + box area) | 37 | 3138.764 |
| 12 | ɸ(~year + FAL)p(~year * region) | 76 | 3138.878 |
| 13 | ɸ(~colony size)p(~year + box area) | 37 | 3138.944 |
| 14 | ɸ(~region * FAL)p(~year + box area) | 39 | 3139.091 |
| 15 | ɸ(~FAL * colony size)p(~year + box area) | 39 | 3139.13 |
| 16 | ɸ(~year)p(~year * region) | 75 | 3140.724 |
| 17 | ɸ(~FAL)p(~year + region) | 35 | 3152.109 |
| 18 | ɸ(~region + FAL)p(~year + region) | 36 | 3153.797 |
| 19 | ɸ(~FAL)p(~year) | 34 | 3153.824 |
| 20 | ɸ(~FAL + colony size)p(~year + region) | 36 | 3153.874 |
| 21 | ɸ(~FAL)p(~year * box area) | 97 | 3154.689 |
| 22 | ɸ(~region + FAL)p(~year) | 35 | 3155.304 |
| 23 | ɸ(~region)p(~year + region) | 35 | 3155.326 |
| 24 | ɸ(~FAL + colony size)p(~year) | 35 | 3155.419 |
| 25 | ɸ(~colony size)p(~year + region) | 35 | 3155.42 |
| 26 | ɸ(~FAL * colony size)p(~year + region) | 37 | 3155.576 |
| 27 | ɸ(~region * FAL)p(~year + region) | 37 | 3155.597 |
| 28 | ɸ(~region + FAL)p(~year * box area) | 98 | 3156.317 |
| 29 | ɸ(~FAL + colony size)p(~year * box area) | 98 | 3156.541 |
| 30 | ɸ(~region)p(~year) | 34 | 3156.809 |
| 31 | ɸ(~FAL)p(~year * box area + effort) | 98 | 3156.814 |
| 32 | ɸ(~colony size)p(~year) | 34 | 3156.944 |
| 33 | ɸ(~region * FAL)p(~year) | 36 | 3157.111 |
| 34 | ɸ(~FAL * colony size)p(~year) | 36 | 3157.127 |
| 35 | ɸ(~region)p(~year * box area) | 97 | 3157.736 |
| 36 | ɸ(~FAL)p(~year + effort) | 35 | 3157.832 |
| 37 | ɸ(~colony size)p(~year * box area) | 97 | 3158.004 |
| 38 | ɸ(~region * FAL)p(~year * box area) | 99 | 3158.172 |
| 39 | ɸ(~FAL * colony size)p(~year * box area) | 99 | 3158.302 |
| 40 | ɸ(~region + FAL)p(~year * box area + effort) | 99 | 3158.42 |
| 41 | ɸ(~FAL + colony size)p(~year * box area + effort) | 99 | 3158.54 |
| 42 | ɸ(~region + FAL)p(~year * effort) | 67 | 3158.906 |
| 43 | ɸ(~colony size)p(~year + effort) | 35 | 3158.924 |
| 44 | ɸ(~FAL + colony size)p(~year * effort) | 67 | 3159.039 |
| 45 | ɸ(~FAL * colony size)p(~year + effort) | 37 | 3159.106 |
| 46 | ɸ(~region + FAL)p(~year + effort) | 36 | 3159.512 |
| 47 | ɸ(~FAL + colony size)p(~year + effort) | 36 | 3159.618 |
| 48 | ɸ(~region)p(~year * box area + effort) | 98 | 3159.792 |
| 49 | ɸ(~colony size)p(~year * box area + effort) | 98 | 3160.005 |
| 50 | ɸ(~FAL)p(~year * effort) | 66 | 3160.01 |
| 51 | ɸ(~region * FAL)p(~year * box area + effort) | 100 | 3160.173 |
| 52 | ɸ(~region)p(~year * effort) | 66 | 3160.35 |
| 53 | ɸ(~FAL * colony size)p(~year * box area + effort) | 100 | 3160.463 |
| 54 | ɸ(~colony size)p(~year * effort) | 66 | 3160.508 |
| 55 | ɸ(~FAL * colony size)p(~year * effort) | 68 | 3160.738 |
| 56 | ɸ(~region)p(~year + effort) | 35 | 3161.047 |
| 57 | ɸ(~region * FAL)p(~year + effort) | 37 | 3161.285 |
| 58 | ɸ(~region * FAL)p(~year * effort) | 68 | 3163.595 |
| 59 | ɸ(~year + FAL)p(~year + box area) | 68 | 3167.022 |
| 60 | ɸ(~year)p(~year + box area) | 67 | 3168.573 |
| 61 | ɸ(~year * FAL)p(~year * region) | 107 | 3178.286 |
| 62 | ɸ(~year + FAL)p(~year + region) | 66 | 3180.096 |
| 63 | ɸ(~year + FAL)p(~year) | 65 | 3181.372 |
| 64 | ɸ(~year)p(~year + region) | 65 | 3182.006 |
| 65 | ɸ(~year)p(~year) | 64 | 3183.286 |
| 66 | ɸ(~year + FAL)p(~year + effort) | 66 | 3183.337 |
| 67 | ɸ(~year)p(~year + effort) | 65 | 3185.211 |
| 68 | ɸ(~year + FAL)p(~year * effort) | 97 | 3187.139 |
| 69 | ɸ(~year + FAL)p(~year * box area) | 128 | 3188.369 |
| 70 | ɸ(~year)p(~year * effort) | 96 | 3188.736 |
| 71 | ɸ(~year)p(~year * box area) | 127 | 3189.96 |
| 72 | ɸ(~year + FAL)p(~year * box area + effort) | 129 | 3190.37 |
| 73 | ɸ(~year)p(~year * box area + effort) | 128 | 3191.961 |
| 74 | ɸ(~year * FAL)p(~year + box area) | 99 | 3206.898 |
| 75 | ɸ(~year * FAL)p(~year + region) | 97 | 3220.037 |
| 76 | ɸ(~year * FAL)p(~year) | 96 | 3221.23 |
| 77 | ɸ(~year * FAL)p(~year + effort) | 97 | 3223.19 |
| 78 | ɸ(~year * FAL)p(~year * effort) | 128 | 3225.755 |
| 79 | ɸ(~year * FAL)p(~year * box area) | 159 | 3225.931 |
| 80 | ɸ(~year * FAL)p(~year * box area + effort) | 160 | 3229.759 |
| 81 | ɸ(~FAL)p(~boxarea) | 6 | 3240.866 |
| 82 | ɸ(~region + FAL)p(~box area) | 7 | 3242.453 |
| 83 | ɸ(~FAL + colony size)p(~box area) | 7 | 3242.604 |
| 84 | ɸ(~region)p(~boxarea) | 6 | 3244.017 |
| 85 | ɸ(~colony size)p(~box area) | 6 | 3244.194 |
| 86 | ɸ(~region * FAL)p(~box area) | 8 | 3244.308 |
| 87 | ɸ(~FAL * colony size)p(~box area) | 8 | 3244.365 |
| 88 | ɸ(~FAL)p(~region) | 4 | 3251.685 |
| 89 | ɸ(~region + FAL)p(~region) | 5 | 3253.314 |
| 90 | ɸ(~FAL + colony size)p(~region) | 5 | 3253.361 |
| 91 | ɸ(~region)p(~region) | 4 | 3254.956 |
| 92 | ɸ(~colony size)p(~region) | 4 | 3255.016 |
| 93 | ɸ(~FAL * colony size)p(~region) | 6 | 3255.128 |
| 94 | ɸ(~region * FAL)p(~region) | 6 | 3255.179 |
| 95 | ɸ(~FAL)p(~effort) | 4 | 3256.957 |
| 96 | ɸ(~region + FAL)p(~effort) | 5 | 3257.9 |
| 97 | ɸ(~FAL + colony size)p(~effort) | 5 | 3258.027 |
| 98 | ɸ(~year + FAL)p(~box area) | 37 | 3259.382 |
| 99 | ɸ(~region)p(~effort) | 4 | 3259.418 |
| 100 | ɸ(~colony size)p(~effort) | 4 | 3259.565 |
| 101 | ɸ(~region * FAL)p(~effort) | 6 | 3259.8 |
| 102 | ɸ(~FAL * colony size)p(~effort) | 6 | 3259.832 |
| 103 | ɸ(~year)p(~boxarea) | 36 | 3260.482 |
| 104 | ɸ(~year + FAL)p(~region) | 35 | 3269.331 |
| 105 | ɸ(~year)p(~region) | 34 | 3270.45 |
| 106 | ɸ(~year + FAL)p(~effort) | 35 | 3270.971 |
| 107 | ɸ(~year)p(~effort) | 34 | 3271.839 |
| 108 | ɸ(~year * FAL)p(~box area) | 68 | 3299.158 |
| 109 | ɸ(~year * FAL)p(~region) | 66 | 3308.692 |
| 110 | ɸ(~year * FAL)p(~effort) | 66 | 3310.411 |

**Tab. S3:** Model parameters of the best model ɸ(~FAL)p(~year*region): Parameter nr. = model parameter number, Parameter = parameter description, Estimate = maximum likelihood estimates

| **Parameter nr.** | **Parameter** | **Estimate** |
| --- | --- | --- |
| 1 | Phi.(Intercept) | -2.57980429 |
| 2 | Phi.size | 0.09635972 |
| 3 | p.(Intercept) | 12.52426769 |
| 4 | p.time1991 | 20.96590099 |
| 5 | p.time1992 | 24.76838139 |
| 6 | p.time1993 | 26.20063297 |
| 7 | p.time1994 | 27.34071902 |
| 8 | p.time1995 | -10.11790519 |
| 9 | p.time1996 | -10.63087764 |
| 10 | p.time1997 | -10.47408559 |
| 11 | p.time1998 | -11.11062346 |
| 12 | p.time1999 | -9.78896001 |
| 13 | p.time2000 | -12.05772005 |
| 14 | p.time2001 | -9.04108832 |
| 15 | p.time2002 | -10.52903270 |
| 16 | p.time2003 | -10.66614971 |
| 17 | p.time2004 | -11.13171446 |
| 18 | p.time2005 | -12.05005026 |
| 19 | p.time2006 | -12.65930540 |
| 20 | p.time2007 | -11.57284307 |
| 21 | p.time2008 | -12.37870493 |
| 22 | p.time2009 | -11.78977948 |
| 23 | p.time2010 | -12.16122038 |
| 24 | p.time2011 | -12.02550372 |
| 25 | p.time2012 | -12.19610116 |
| 26 | p.time2013 | -13.17977519 |
| 27 | p.time2014 | -11.00623701 |
| 28 | p.time2015 | -11.17577095 |
| 29 | p.time2016 | -10.61095531 |
| 30 | p.time2017 | -11.08079943 |
| 31 | p.time2018 | -9.63118972 |
| 32 | p.time2019 | -7.52639176 |
| 33 | p.time2020 | -9.69226957 |
| 34 | p.time2021 | -9.84432282 |
| 35 | p.regionWB | 9.06188927 |
| 36 | p.time2012:regionWB | 21.32627520 |
| 37 | p.time2013:regionWB | 21.27190081 |
| 38 | p.time2014:regionWB | 31.69895633 |
| 39 | p.time2015:regionWB | -6.50120964 |
| 40 | p.time2016:regionWB | -8.59250872 |
| 41 | p.time2017:regionWB | -8.30945583 |
| 42 | p.time2018:regionWB | -11.42536238 |
| 43 | p.time2019:regionWB | -12.30597120 |
| 44 | p.time2020:regionWB | -8.94991641 |
| 45 | p.time2021:regionWB | -9.15055016 |

**Tab. S4**: Results for estimated recapture probability (estimated p) by year and region, lower confidence level (lcl) and upper confidence level (ucl) from the model ɸ(~FAL)p(~year*region).

| **year** | **region** | **estimate** | **lcl** | **ucl** |
| --- | --- | --- | --- | --- |
| 2021 | NSH | 0.936 | 0.798 | 0.982 |
| 2021 | WB | 0.93 | 0.685 | 0.988 |
| 2020 | NSH | 0.944 | 0.883 | 0.974 |
| 2020 | WB | 0.95 | 0.857 | 0.984 |
| 2019 | NSH | 0.993 | 0.954 | 0.999 |
| 2019 | WB | 0.852 | 0.736 | 0.923 |
| 2018 | NSH | 0.948 | 0.895 | 0.975 |
| 2018 | WB | 0.629 | 0.507 | 0.737 |
| 2017 | NSH | 0.809 | 0.729 | 0.869 |
| 2017 | WB | 0.9 | 0.786 | 0.957 |
| 2016 | NSH | 0.871 | 0.794 | 0.923 |
| 2016 | WB | 0.915 | 0.814 | 0.964 |
| 2015 | NSH | 0.794 | 0.711 | 0.858 |
| 2015 | WB | 0.98 | 0.874 | 0.997 |
| 2014 | NSH | 0.82 | 0.724 | 0.888 |
| 2014 | WB | 1 | 0 | 1 |
| 2013 | NSH | 0.342 | 0.251 | 0.446 |
| 2013 | WB | 1 | 0 | 1 |
| 2012 | NSH | 0.581 | 0.471 | 0.684 |
| 2012 | WB | 1 | 0 | 1 |
| 2011 | NSH | 0.622 | 0.511 | 0.722 |
| 2010 | NSH | 0.59 | 0.476 | 0.694 |
| 2009 | NSH | 0.676 | 0.567 | 0.769 |
| 2008 | NSH | 0.536 | 0.431 | 0.638 |
| 2007 | NSH | 0.721 | 0.609 | 0.811 |
| 2006 | NSH | 0.466 | 0.356 | 0.58 |
| 2005 | NSH | 0.616 | 0.506 | 0.716 |
| 2004 | NSH | 0.801 | 0.69 | 0.879 |
| 2003 | NSH | 0.865 | 0.752 | 0.931 |
| 2002 | NSH | 0.88 | 0.729 | 0.953 |
| 2001 | NSH | 0.97 | 0.819 | 0.996 |
| 2000 | NSH | 0.615 | 0.459 | 0.75 |
| 1999 | NSH | 0.939 | 0.792 | 0.984 |
| 1998 | NSH | 0.804 | 0.634 | 0.907 |
| 1997 | NSH | 0.886 | 0.709 | 0.961 |
| 1996 | NSH | 0.869 | 0.706 | 0.948 |
| 1995 | NSH | 0.917 | 0.775 | 0.973 |
| 1994 | NSH | 1 | 0 | 1 |
| 1993 | NSH | 1 | 0 | 1 |
| 1992 | NSH | 1 | 0 | 1 |
| 1991 | NSH | 1 | 0 | 1 |
| 1990 | NSH | 1 | 0 | 1 |

**Tab. S5**: Results for estimated survival probabaility (estimated ɸ) depending on forearm length (FAL, in mm), lower confidence level (lcl) and upper confidence level (ucl) from the model ɸ(~FAL)p(~year*region).

| **FAL** | **estimated ɸ** | **lcl** | **ucl** |
| --- | --- | --- | --- |
| 36.9 | 0.726 | 0.671 | 0.775 |
| 37.0 | 0.728 | 0.675 | 0.776 |
| 37.1 | 0.73 | 0.679 | 0.776 |
| 37.2 | 0.732 | 0.682 | 0.777 |
| 37.4 | 0.736 | 0.689 | 0.778 |
| 37.6 | 0.739 | 0.696 | 0.779 |
| 37.7 | 0.741 | 0.7 | 0.779 |
| 37.8 | 0.743 | 0.703 | 0.78 |
| 37.9 | 0.745 | 0.706 | 0.78 |
| 38.0 | 0.747 | 0.71 | 0.781 |
| 38.1 | 0.749 | 0.713 | 0.781 |
| 38.2 | 0.75 | 0.716 | 0.782 |
| 38.3 | 0.752 | 0.72 | 0.782 |
| 38.4 | 0.754 | 0.723 | 0.783 |
| 38.5 | 0.756 | 0.726 | 0.783 |
| 38.6 | 0.758 | 0.729 | 0.784 |
| 38.7 | 0.759 | 0.732 | 0.785 |
| 38.8 | 0.761 | 0.735 | 0.785 |
| 38.9 | 0.763 | 0.738 | 0.786 |
| 39.0 | 0.765 | 0.741 | 0.787 |
| 39.1 | 0.766 | 0.744 | 0.787 |
| 39.2 | 0.768 | 0.747 | 0.788 |
| 39.3 | 0.77 | 0.75 | 0.789 |
| 39.4 | 0.771 | 0.752 | 0.789 |
| 39.5 | 0.773 | 0.755 | 0.79 |
| 39.6 | 0.775 | 0.758 | 0.791 |
| 39.7 | 0.777 | 0.76 | 0.792 |
| 39.8 | 0.778 | 0.763 | 0.793 |
| 39.9 | 0.78 | 0.765 | 0.794 |
| 40.0 | 0.782 | 0.767 | 0.795 |
| 40.1 | 0.783 | 0.769 | 0.797 |
| 40.2 | 0.785 | 0.771 | 0.798 |
| 40.3 | 0.786 | 0.773 | 0.799 |
| 40.4 | 0.788 | 0.775 | 0.801 |
| 40.5 | 0.79 | 0.776 | 0.803 |
| 40.6 | 0.791 | 0.778 | 0.804 |
| 40.7 | 0.793 | 0.779 | 0.806 |
| 40.8 | 0.794 | 0.78 | 0.808 |
| 40.9 | 0.796 | 0.781 | 0.81 |
| 41.0 | 0.798 | 0.782 | 0.812 |
| 41.1 | 0.799 | 0.783 | 0.814 |
| 41.2 | 0.801 | 0.784 | 0.816 |
| 41.3 | 0.802 | 0.785 | 0.818 |
| 41.4 | 0.804 | 0.786 | 0.82 |
| 41.5 | 0.805 | 0.787 | 0.822 |
| 41.6 | 0.807 | 0.787 | 0.825 |
| 41.7 | 0.808 | 0.788 | 0.827 |
| 41.8 | 0.81 | 0.789 | 0.829 |
| 41.9 | 0.811 | 0.789 | 0.831 |
| 42.0 | 0.813 | 0.79 | 0.833 |
| 42.1 | 0.814 | 0.791 | 0.836 |
| 42.2 | 0.816 | 0.791 | 0.838 |
| 42.3 | 0.817 | 0.792 | 0.84 |
| 42.4 | 0.818 | 0.792 | 0.842 |
| 42.5 | 0.82 | 0.793 | 0.844 |
| 42.6 | 0.821 | 0.793 | 0.846 |
| 42.7 | 0.823 | 0.794 | 0.848 |
| 42.8 | 0.824 | 0.794 | 0.85 |
| 42.9 | 0.825 | 0.795 | 0.852 |
| 43.1 | 0.828 | 0.796 | 0.856 |
| 43.5 | 0.834 | 0.798 | 0.864 |

## Colony size

The number of individuals in a colony in a given year (colony size) was calculated as the number of all adult individuals captured, plus the number of all adult individuals that were not captured, but alive, as they had been recaptured in a later year. On the first capture occasion and if the recapture rate of individuals of a specific colony in a given year dropped below 70 %, we interpolated colony size in this specific year using the *na_ma* function (weighted moving average) of the package imputeTS (Moritz 2017). Moreover, high proportion of unmarked adult bats and the strongly increasing colony sizes during first years in the colonies WT1, WT2, obererTUP, untererTUP and WB indicated a bad coverage of the colonies during the first years. Therefore, we also interpolated the colony size for the first years in these colonies (see green dots in Fig. S3). We then calculated the mean colony size for each colony across the years.


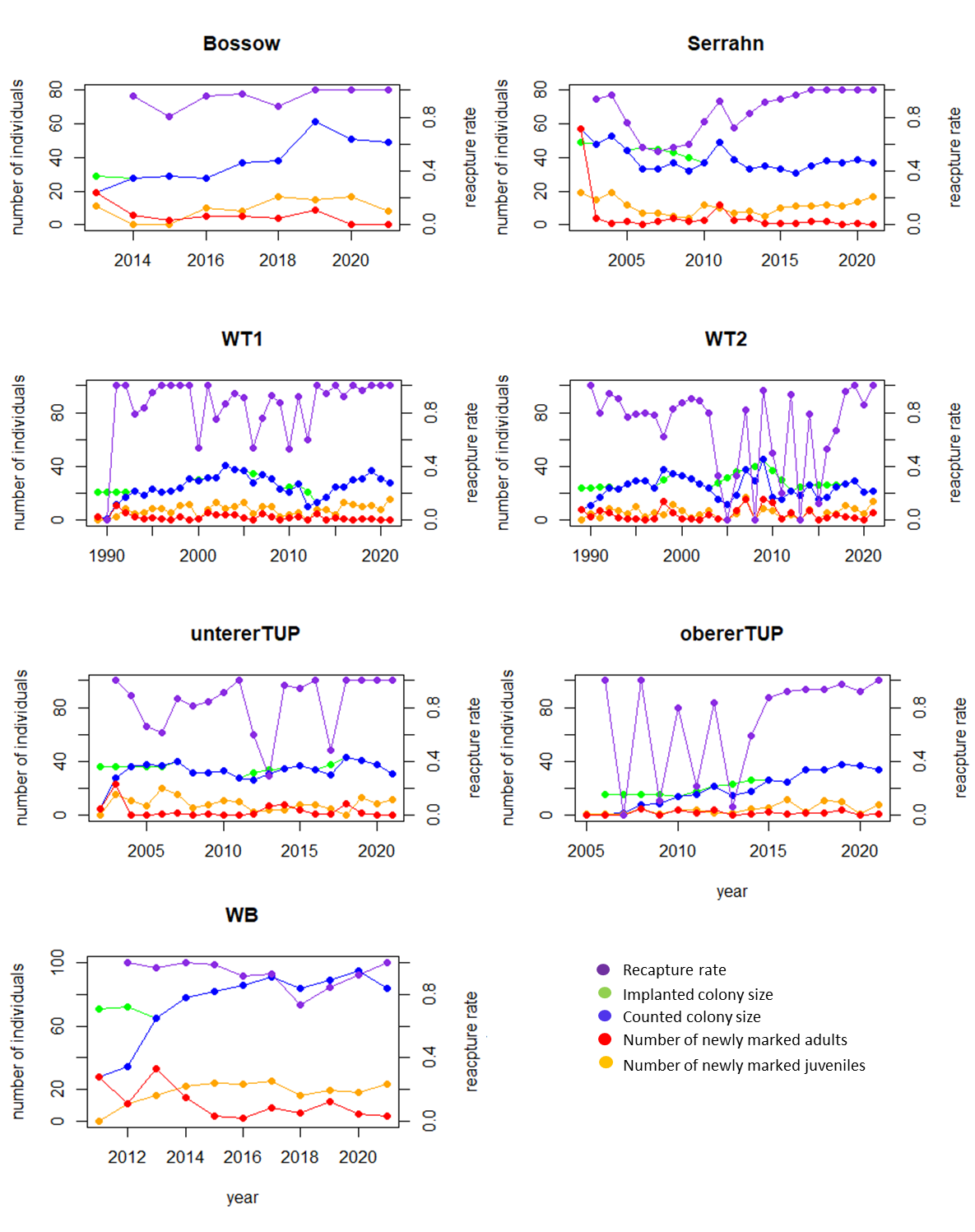


**Fig. S3:** Overview of recapture rate (violet, right y-axis), interpolated colony size (green, left y-axis), counted colony size (blue, left y-axis), number of newly marked adult individuals (red, left y-axis) and number of newly marked juvenile individuals (yellow, left y-axis) for each year.
